# A full response chain surge capacity test of a small rural hospital, prehospital resources and collaborating organisations

**DOI:** 10.1186/s13049-025-01372-9

**Published:** 2025-03-28

**Authors:** Kristina Stølen Ugelvik, Kristina Lennquist Montán, Øyvind Thomassen, Geir Sverre Braut, Thomas Geisner, Silje Longva Todnem, Ove Njå, Elin Seim, Torunn Oveland Apelseth, Janecke Engeberg Sjøvold, Geir Arne Sunde, Sølvi Kasin, Carl Montán

**Affiliations:** 1 Regional Trauma Centre, Haukeland University Hospital, Bergen, Norway; 2 University of Bergen, Bergen, Norway; 3 Department of Global Public Health, Karolinska Institute, Stockholm, Sweden; 4 Haukeland University Hospital, Bergen, Norway; 5 Norwegian Air Ambulance Foundation, Drøbak, Norway; 6 Stavanger University Hospital, Stavanger, Norway; 7 Western Norway University of Applied Sciences, Bergen, Norway; 8 Gastrointestinal Surgery Department, Haukeland University, Bergen, Norway; 9 Hardanger and Voss Emergency Primary Health Care Service, Bergen, Norway; 10 University of Stavanger, Stavanger, Norway; 11 Emergency Department, Voss Hospital, Bergen, Norway; 12 Department of Immunology and Transfusion Medicine, Haukeland University Hospital, Bergen, Norway; 13 Institute of Clinical Science, University of Bergen, Bergen, Norway; 14 University of Tromsø, Tromsø, Norway; 15 Department of Molecular Medicine and Surgery, Karolinska Institutet, Stockholm, Sweden

**Keywords:** Major incident, Mass casualty incident, Surge capacity, Mass casualty incident simulation, Hospital preparedness, Surge capacity test

## Abstract

**Background:**

Increased surge capacity is key in mass casualty incidents. Rural hospitals face other challenges in terms of transport capacity and available resources. The aim was to examine if a simulation system previously used to test surge capacity at large hospitals, could be used to test surge capacity at a small rural hospital.

**Method:**

A qualitative study was conducted to assess surge capacity at a small rural hospital using a previously validated simulation system. The simulation system was adopted to the Norwegian trauma system and local context. New simulated patient cards were developed, inspired by traffic victims. A tunnel accident scenario involving a bus, a heavy goods vehicle and a motorcyclist was used. Test staff ensured that real consumption of time and resources were followed. 98 persons representing 16 organisations, participated. A post-test survey was collected.

**Results:**

Access to the scene and transport resources were bottlenecks in the initial phase. The emergency department and lack of surgeons and anaesthetic doctors in the trauma team became the first and most prominent in-hospital surge capacity limiting factors. Operating theatre reached surge capacity, but never exceeded. The intensive care unit avoided depletion of beds/staff/ventilators due to transfer of patients to the trauma centre. Surge capacity was enhanced by obtaining staff, blood and equipment from the trauma centre. Water lock systems and replenishment routines for chest tube trays was inadequate. Blood supply was insufficient in the initial phase and a lack of overview of blood products was identified. Some communication gaps and deficiencies in victim identification were detected. The hospital participants evaluated the method as useful in assessing hospital surge capacity. Half of the participants requested increased time to learn the system pre-test. The inclusion of several organisations in the mass casualty incident exercise was appreciated and ranked high as a simulation training.

**Conclusion:**

The simulation system provided detailed data to determine surge capacity and capacity-limiting factors in the mass casualty incidents response at a rural hospital and performed as a training tool for staff. Methods to improve pre-test simulation system knowledge should be examined. Broad inclusion of cooperating organisations was found beneficial.

**Supplementary Information:**

The online version contains supplementary material available at 10.1186/s13049-025-01372-9.

## Introduction

Improving hospital preparedness for mass casualty incidents (MCI) and major incidents (MI) is a crucial part of disaster resilience [1]. Experiences from previous terrorist attacks in Europe indicate that hospitals located near the incident are likely to receive a high number of casualties, mandating that hospitals need to be prepared for these rare, but potentially complex events [2–4].

There are several attempts to define surge capacity (SC) in literature, but no prevailing definition exists [5, 6]. Stratton et al. define SC as the ability to manage a sudden increase in the number of patients after a disaster or an emergency, as opposed to daily patient care [7]. Hick et al. suggest the 4 S`s as key factors in SC; “system, space, staff, and supplies” [5]. Differences in taxonomy and lack of standardised benchmarks for assessing SC, complicates the comparison of hospitals in terms of data and contingency framework. However, it is undisputed that knowledge about the hospital’s SC, capacity-limiting factors and strategies to increase SC is crucial when the disaster hits [8–10]. Different methods to assess and enhance SC have been suggested [11–13]. The Mass Casualty Simulation System (MACSIM^®^^)^ simulation system was developed to compare MCI/MI triage methods and was further used to train participants “full-chain” in a post-graduate course, Medical Response to Major Incident (MRMI) [14, 15]. The MACSIM^®^ system has also been applied as a method to examine hospital SC at 3 larger Swedish hospitals [16, 17]. SC testing by the MACSIM^®^ methodology has never been conducted in Norway and never at a hospital with < 300 beds-capacity.

**Fig. 1:**
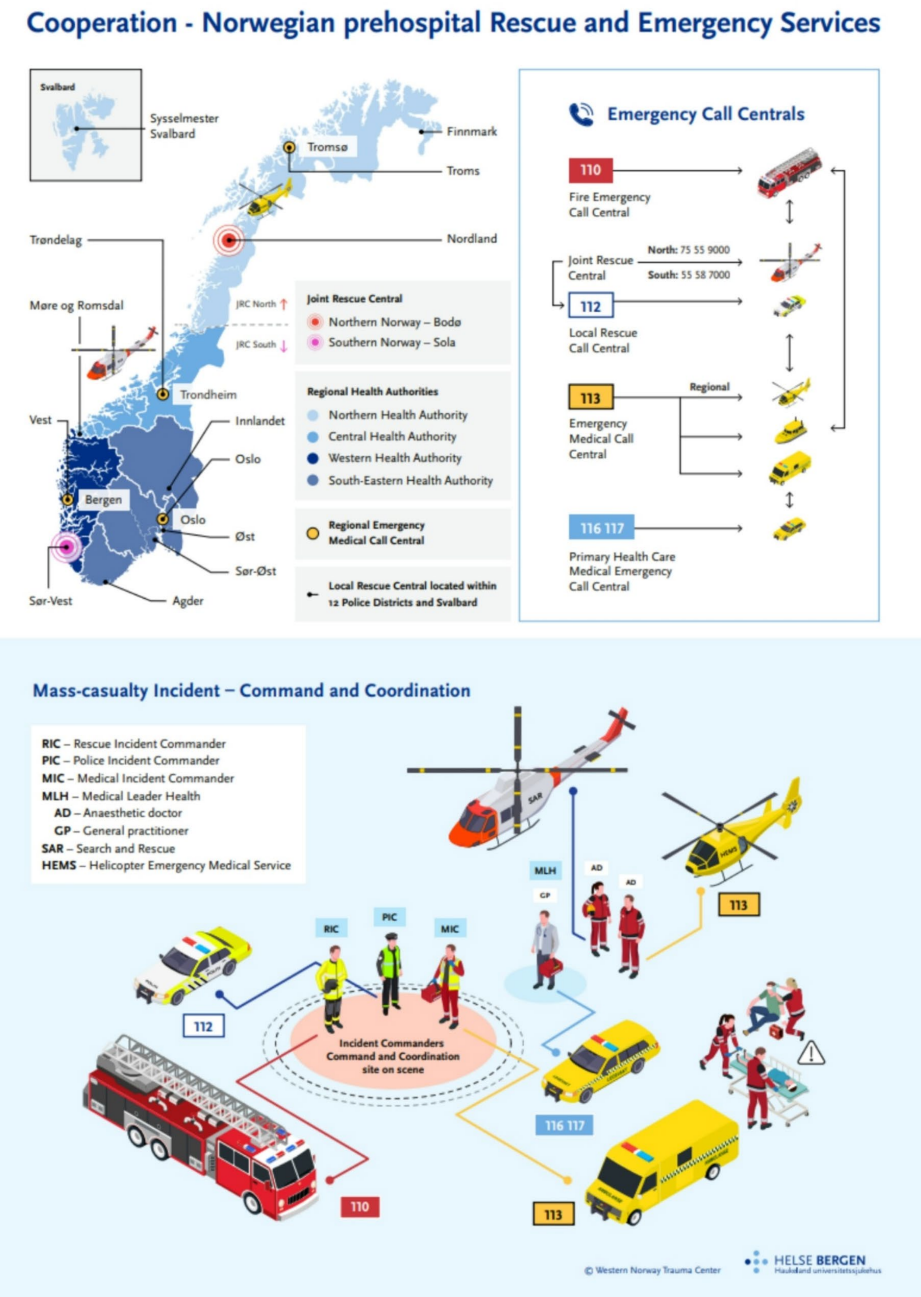
Norwegian prehospital MCI/MI organisation

Bachmann et al. identified communication, triage and transport as 3 out of 4 key elements in MCI/MI preparedness [18]. Norwegian national guidelines describe prehospital cooperation, communication lines and responsibilities [19, 20]. Figure 1 illustrate the Norwegian prehospital system as described in the article by Ugelvik et al. [21]. The national trauma plan defines patient flow to acute hospitals with trauma function (NTC) and trauma centres (TC) [22]. Traffic accidents and high energy falls are the most common causes of trauma in Norway [23].

Voss Hospital (VH) is an NTC with 64-beds capacity, located in the western part of Norway 100 km from the TC, Haukeland University Hospital (HUH) [22]. The trauma volume is low [23]. Compared to HUH, VH faces several limitations in terms of space, staff and supplies. Transport resources in the region are likely to be constrained in an MCI/MI, especially in adverse weather conditions. There are several single-tube road tunnels with bi-directional traffic and longitudinal ventilation in the region. Steep, narrow, bending roads frequently experiencing rockfalls, snow avalanches and landslides onto/over the roads. Consequently, patient transport and patient flow are not only influenced by triage, coordination and communication, but also by environmental conditions. Cooperation with HUH is crucial in terms of patient referral and possible enhancement of SC, as staff and supplies can be distributed from HUH. An intermunicipal out-of-hours emergency primary health care service (OOH) is also located at VH and operative between 4:00 pm-8:00 am on weekdays and throughout the weekends. Being a rural hospital with inherent limitations in SC, put coordination, communication and cooperation to the test.

The aim of this study was to investigate if a simulation model could be used to assess SC and capacity-limiting factors at a small rural hospital, conducting a full chain MCI exercise involving relevant civilian organisations to test transport resources and cooperation.

## Method

A qualitative study to assess SC and capacity-limiting factors at a small rural hospital using the MACSIM^®^ methodology was conducted. Qualitative and quantitative data were collected and analysed.

### Planning phase

**Fig. 2:**
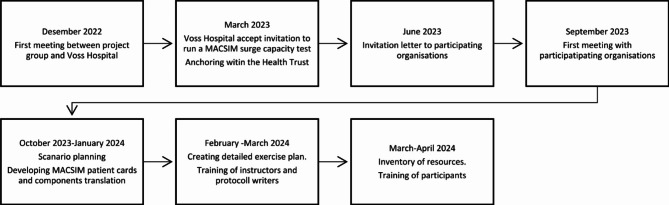
Timeline planning

**Fig. 3:**
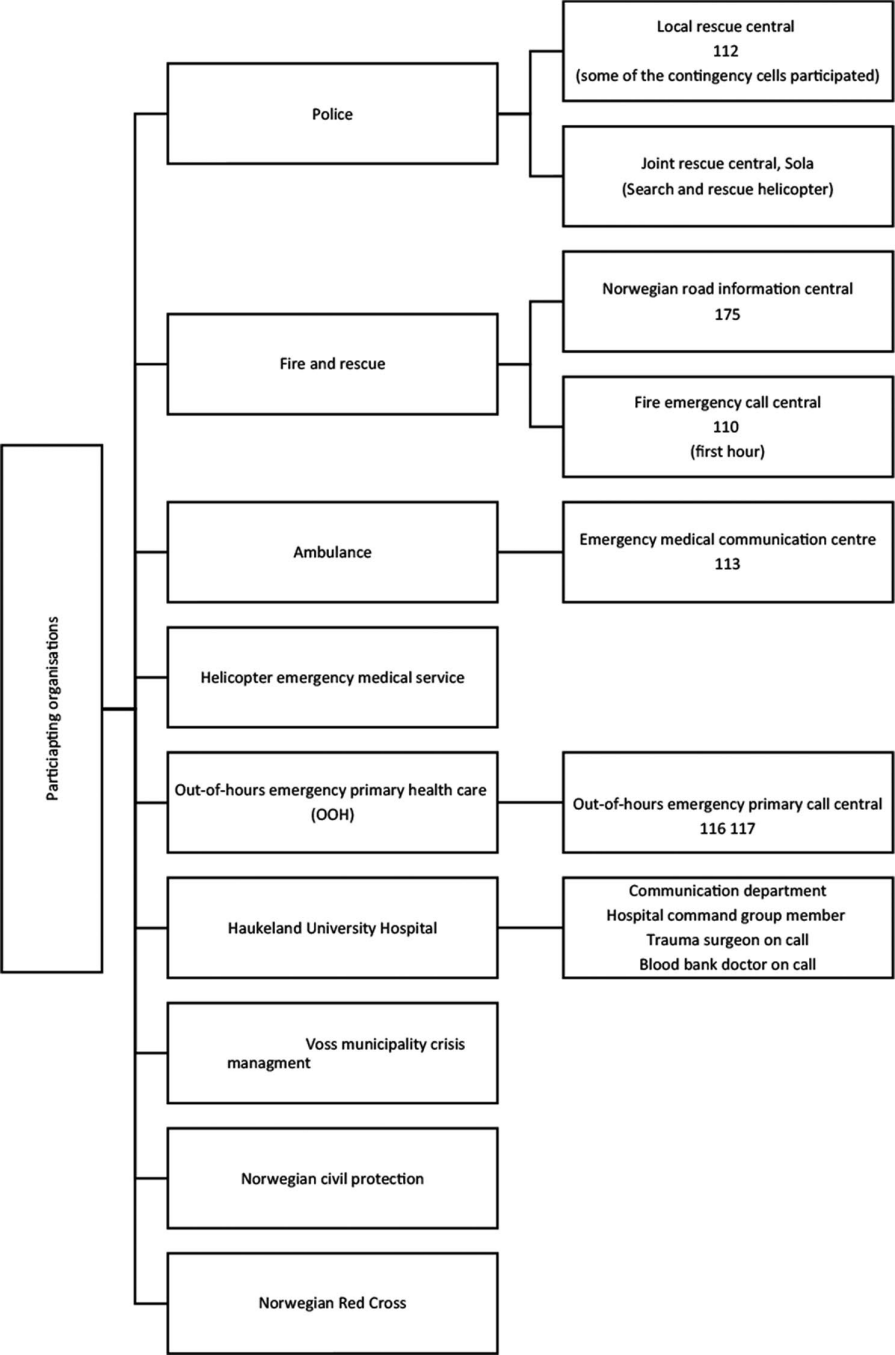
Participating organisations in the “Vossa-test” (excluding Voss Hospital)

Early leadership commitment and local involvement were aimed for (Fig. 2). The SC test called “Vossa-test”, was designed as an MCI/MI exercise for non-medical participants to test communication, collaboration, transport capacity, media/social media management and patient identification (Fig. 3). In addition to the general aims, each participating organisation was encouraged to define their own specific learning goals (Table 1).

**Table 1:** Learning goals for the “Vossa-test”

**Overall aims:**
Test the existing contingency plans
Train staff in decision-making, collaboration and communication during an MCI/MI
**Specific aims**:
Test the MCI/MI transport capacity, with the existing geographical and environmental conditions
Perform a surge capacity test at Voss Hospital
Test the blood supply chain
Train the participating staff in their roles during an MCI/MI
Train participants in communication and media/social media management during an MCI/MI
Identification of patients during an MCI/MI

Tunnel security experts and local tunnel construction personnel contributed to the development of the scenario. The Stalheim tunnel, characterised by its steep and bending structure and lack of escape channel, served as the site of the accident. The tunnel accommodates heavy goods transport and has seasonal high volume of bus tourism. The scenario involved a rock avalanche blocking the tunnel exit. A heavy goods vehicle (HGV) marked with general warning signal for dangerous goods (lithium batteries) failed to stop in time and collided with the avalanche. This collision was followed by a bus carrying elderly German tourists crashing into the HGV and a motorcyclist who struck the bus resulting in 60 casualties. A detailed exercise plan was created to ensure coordination among instructors.

The MACSIM^®^ system is described in detail by Lennquist Montán et al. [15–17]. The table-top system consists of magnetic boards with the available physical spaces, personnel categories, transport resources, patient cards and treatment tags indicating time consumed (Additional file 1). The components of the MACSIM^®^ simulation system were translated to Norwegian and adapted to the Norwegian trauma system. New patient cards inspired by traffic accident victims were produced. The realism of each patient case, decisions about timelines for action, optimal treatment and consequences of delayed or lack of optimal treatment was set by the expert opinions of some of the co-authors with advice from different medical specialists. Instructors, protocol writers and participants received simulation system training before the SC test.

An inventory for supplies was conducted 1–3 months prior to the test. Activation of an MCI/MI test alarm on March 20, 2024 (5:30 pm), served as a base for medical staff availability during the test. Transport resources and bed capacity was based on actual occupancy rate on April 15, 2024. Blood supply inventory was gathered on April 16, 2024.

### Conduct phase

A total of 98 individuals participated in the “Vossa-test” on April 17, 2024. The test staff consisted of 1 SC test leader, 26 instructors, 12 protocol writers and 11 evaluators. While the “Vossa-test” was conducted during office hours, the scenario itself was set out of office hours (5:30 pm) to include the OOH. The national radio communication system and a phone directory were readily available to all participants. Participants wore uniforms and brought their emergency equipment. Phone calls, media and social media messages were distributed at predefined times.

The emergency services had to evaluate the risk by gathering information about the tunnel and the heavy goods vehicle load. Victims had to be evacuated through the tunnel entrance. Some of the victims had self-evacuated by foot or by private cars to the tunnel entrance and some to VH in private cars. Instructors challenged the non-hospital participants in terms of MCI/MI risk analysis and communication. The exercise plan described dynamic changes in environmental conditions that affected helicopter and road transport. Real-time data was used to determine transport times to and from the scene of the accident and between hospitals.

The participants had to make active decisions to empty departments of non-trauma cases. In accordance with previous MACSIM^®^ SC tests, instructors were equipped with supplementary information including X-rays, lab results and surgical findings for each patient [16, 17]. Instructors ensured that necessary resources to examine and treat patients were adhered, that predefined consumed time on procedures and actions were followed, updated changes in patient conditions and declared deaths when predefined time-limits were exceeded.

Debriefings were held at the test-stations followed by a joint gathering. Feedback has been provided orally to the hospital management combined with a detailed SC evaluation report.

### Analysis phase

Several methods were used to gather qualitative and quantitative test data.

- Patient cards using MACSIM^®^ methodology as elucidated by Lennquist Montán et al. [15–17].
  - Protocol writers recorded time data at the test-stations.
  - The MACSIM^®^ patient cards were collected after the test, with all treatment and investigation tags left on the card (Additional file 1).
- Logs provided qualitative data regarding timelines and decisions.
  - Exercise plan.
  - Evaluator logs.
  - Emergency medical communication centre log.
  - Hospital command group (HCG) log.
  - Oral debriefs notes.
- Participants evaluation.
  - The software Webropol (Webropol, Helsinki, Finland) was used to collect evaluation from participants.
  - The web-based survey consisted of a maximum of 30 questions depending on the employee’s organisation. Only health personnel were asked to evaluate the MACSIM^®^ methodology as a surge capacity assessment tool (Additional file 2).
  - Data was collected from 19.04.2024–17.05.2024.

### Statistics

Microsoft Office 365 Excel Version 16.0 (Microsoft Corporation, Redmond, WA, US) and SPSS Statistics for Windows, Version 29.0 (IBM, New York, NY, US) were used for analysis. Percentages, median and IQR were applied for descriptive statistics.

## Results

### Running the test

#### Prehospital

**Fig. 4:**
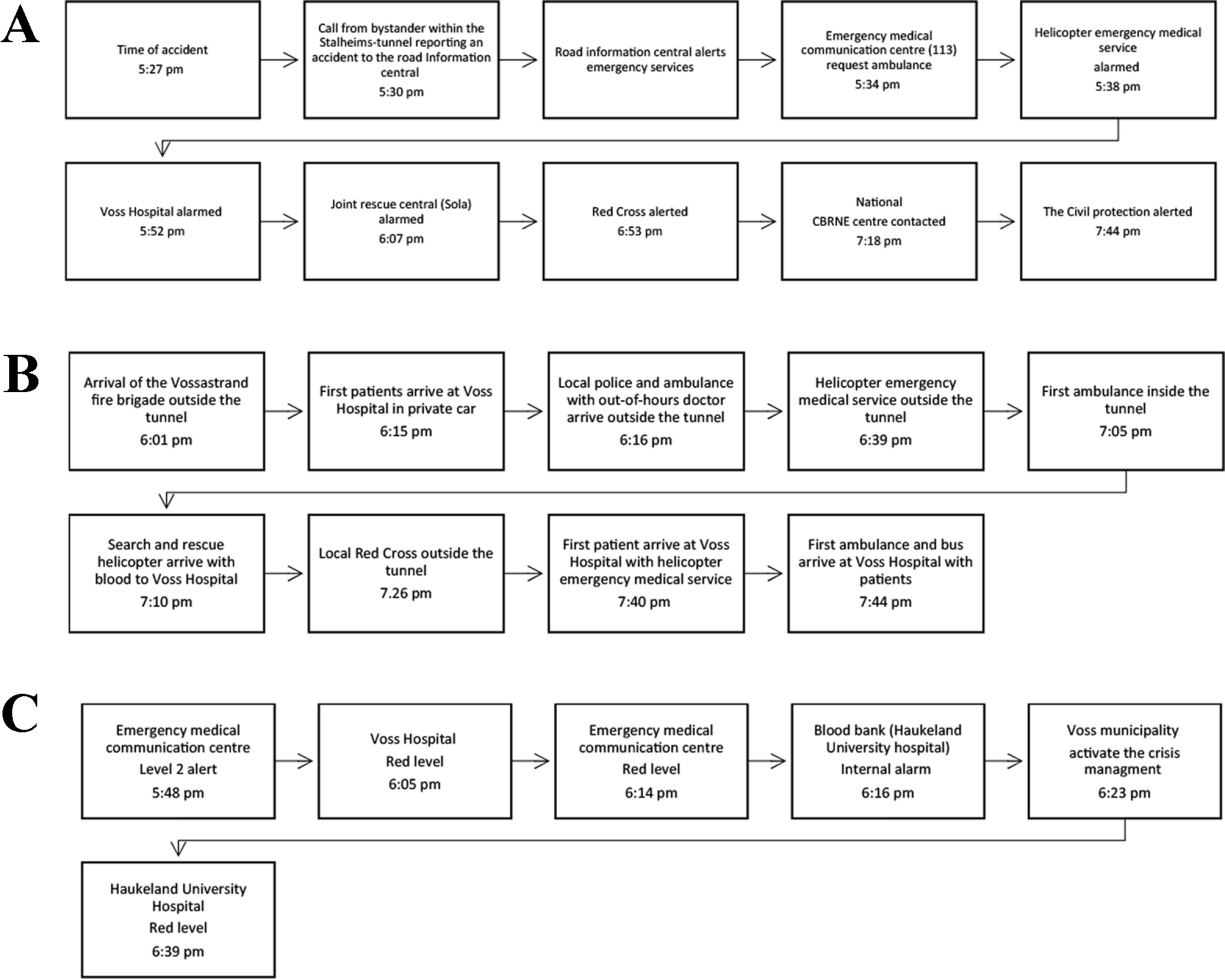
(**A**) Timeline alarming. (**B**) Timeline arrival. (**C**) Activation of disaster levels during the “Vossa-test”

The road information central received a phone call from within the Stalheim tunnel, reporting an accident (Fig. 4A). The first response unit arriving at the scene was the fire brigade, describing chaos outside the tunnel and estimated 32 victims (Fig. 4B). A decision was made to await more resources before entering the tunnel. Activation of red alert level followed (Fig. 4C).

The OOH arrived with the first ambulance and functioned as medical leader health. An incident command post was established. The first arriving anaesthetic doctor from the helicopter emergency medical service (HEMS) prioritised and treated patients, instead of transporting patients one by one. Due to fear of explosion, the ambulances were delayed access to victims inside the tunnel. After access, a suspicion of leakage from lithium batteries resulted in tunnel evacuation and the establishment of a 300-meter hot zone, before the tunnel was finally reopened. Different modes of patient transport were used (Table 2).

**Table 2:** Patient transport from the scene of incident

Transport mode	Number
Private car	13
Helicopter (HEMS/SAR)	5
Ambulance	26
Bus	5
Police car	2
Fire vehicle	1
Out-of-hours emergency primary health care vehicle	1

#### In-hospital triage

**Fig. 5:**
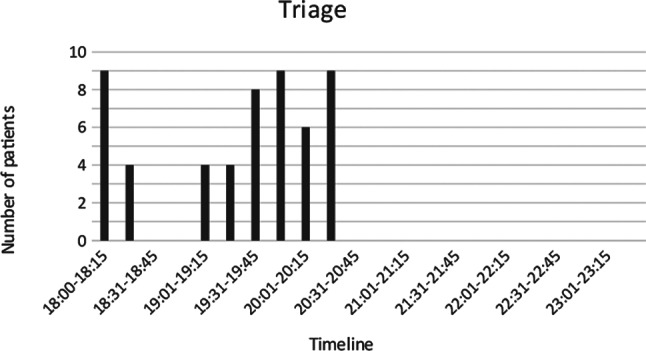
Timeline for patients arriving in triage area at Voss Hospital

**Fig. 6:**
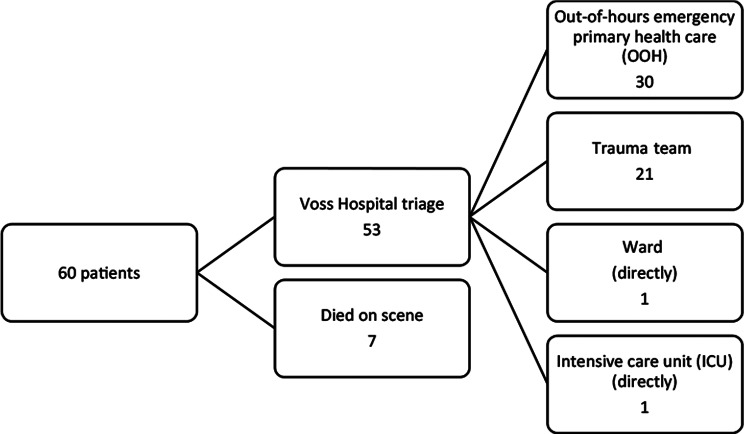
Patient flow from scene and in-hospital triage at Voss Hospital

**Fig. 7:**
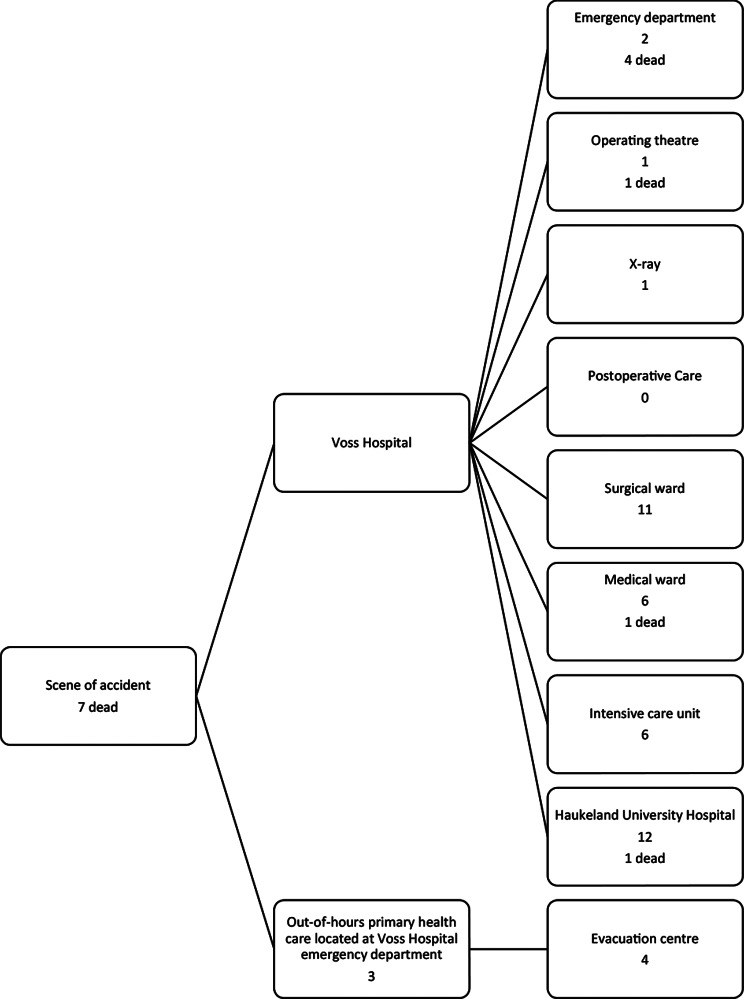
Patient distribution by end of the test and location for deaths

Triage was performed by a surgeon and partially by OOH (Table 3; Figs. 5, 6 and 7). Undertriage was found to be low, 3/53 (5,7%). Of the “category-red” patients, 6/17 (35,3%) had injury severity score (ISS) < 15 and the average ISS was 24,4 (4–57) (Table 3). One patient was triaged blue.

**Table 3:** In-hospital triage and injury severity score

Triage tag	Total number	Injury severity score (ISS) < 9	Injury severity score (ISS) 9–15	Injury severity score (ISS) > 15
Green	18	15	2	1
Yellow	17	13	2	2
Red	17	3	3	11
Blue	1	0	0	1

Green = Non urgent, minor injuries, Yellow = Urgent, moderate injuries, Red = Immediate, Major life-threatening injuries, Blue = Expectancy, life-threatening injuries with expected small survivor chances with the resources available

### Surge capacity

#### System

External communication and collaboration were generally good, supported by established relationships and participants being used to collaborating. Some challenges in delayed and missed communication and victim identification were detected. Examples of communication challenges included decision regarding tunnel access for health, when the last patient left the scene of the incident and clarification related to CBRNE threat.

#### Space

Measures to enhance SC were initiated when the red alert was activated. In total, 13 patients were transferred to HUH by helicopter (Fig. 7). The test provided detailed information regarding the transferred patients and need for ventilators, surgery and radiology.

**Fig. 8:**
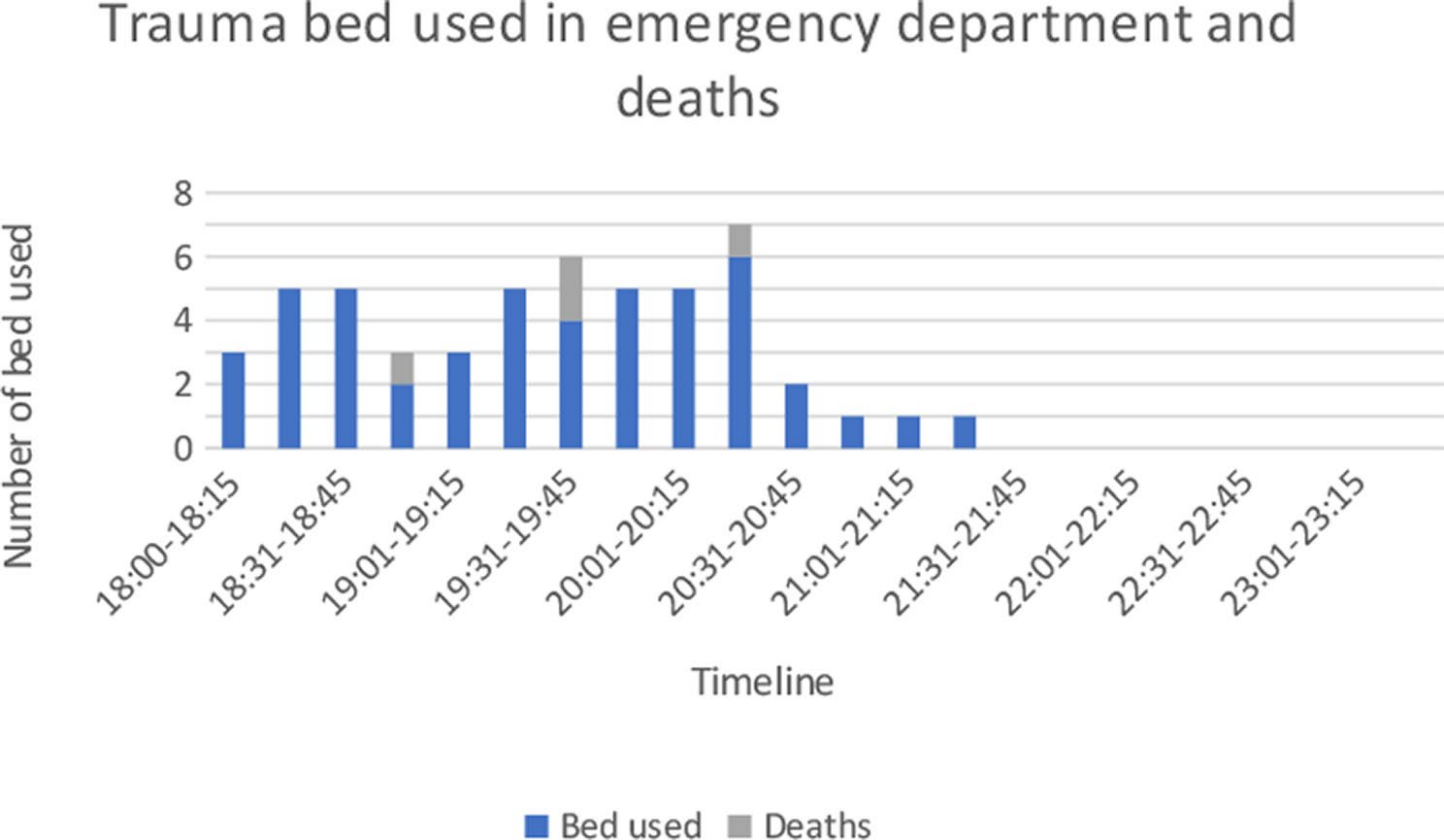
Bed occupancy for trauma in emergency department. Excluding one patient triaged yellow without the need of trauma team and patients managed by out−of−hours primary health care (OOH). Includes two patients that were initially triaged for OOH, but was retriaged as red. Timeline includes waiting time for examination and examination time

In the ED trauma room, a maximum of 3 reduced trauma teams could work simultaneously. Additionally, hospital doctors used 2 rooms and the OOH 8 rooms in the ED. The ED trauma room became a bottleneck in the initial phase (Fig. 8). SC was reached and exceeded for patients triaged red. The waiting time between triage and examination was on average 6 (2-16) minutes for red patients. Average time used for patient examinations in ED (excluding OOH), were 16 (2-55) minutes and 17 (9-29) minutes for red patients. Four patients died in the ED (Fig. 8).

**Fig. 9:**
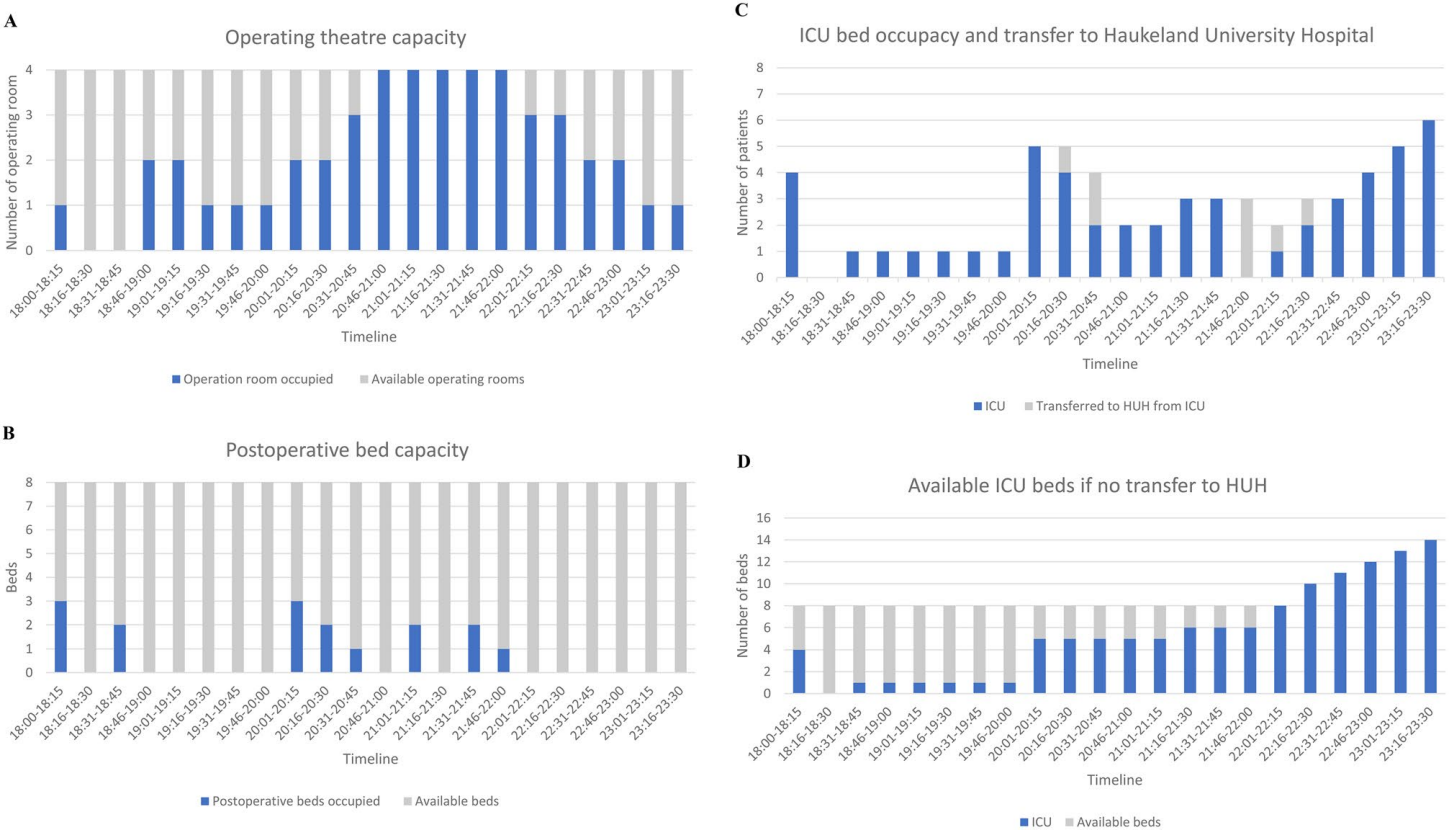
(**A**) Operating theatre capacity. (**B**) Bed occupancy postoperative care. (**C**) Bed occupancy intensive care unit. (**D**) Bed capacity intensive care unit, if no transfer

The operating theatre (OR) reached SC, but did not exceed (Fig. 9A). No immediate patients were delayed for surgery due to lack of OR. Laparotomy was the dominating surgical procedure (Table 4). One patient died after arrival in the OR due to abdominal bleeding and one laparotomy was negative. By test-end, four patients were still waiting for surgery to avoid loss of function or complications. The OR would have been under further constrain if more patients had arrived VH alive and if patient transfer to HUH had not been possible.

**Table 4:** Surgical procedures performed in the operating theatre

Procedure	Number of procedures
Laparotomy	6
Orthopaedic revision of amputated overarm	1

The intensive care unit (ICU) and post operative care (PO) each have an 8-bed capacity. The PO capacity remained adequate and underutilised throughout the test (Fig. 9B). The ICU experienced two peaks (Fig. 9C). Without patient transfer to HUH, the ICU would have surpassed SC (Fig. 9D). Ward capacity was sufficient as patients not related to the event were discharged, moved to gynaecological wards or to a nursing home. Radiology was ordered on paper and limited by one CT scan (Table 5). Challenges were noted in prioritising radiology referrals.

**Table 5:** Radiology

Location	Available radiology modality	Number of availably radiology modality	Examination	Number of examinations performed
Emergency department				
	Ultrasound (US)	1	FAST ^1^	8
	Portable X-ray	1	Chest X-ray	11
			Pelvic X-ray	8
Radiology department				
	Computer tomography (CT)	1	CT trauma^2^	4
			CT head	5
			CT cervical columna	2
			CT total columna	1
	X-ray	3	Chest X-ray	8
			Skeletal X-ray	12
	Magnetic resonance imaging (MRI)	1		
	Ultrasound (US)	1		

*Note* 1. FAST= Focused assessment with sonography in trauma 2. CT trauma protocol: head, neck, thorax, abdomen and pelvis

#### Staff

**Fig. 10:**
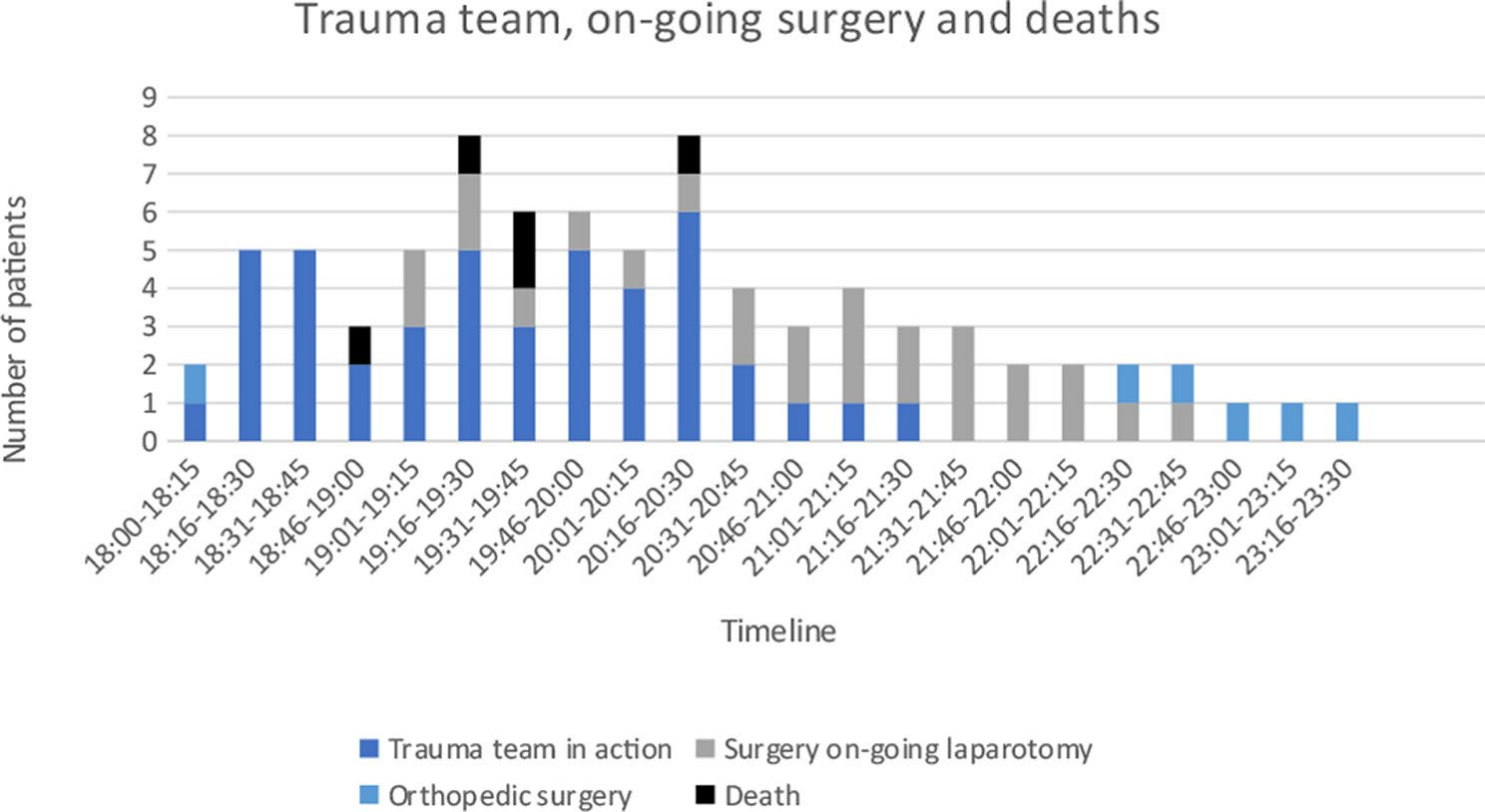
Trauma team, on-going surgery and deaths. Trauma team examining patients (excluding waiting time). Time for surgery includes only knife−time

The number of surgeons in the surgical trauma team was a main limiting factor and SC was exceeded (Fig. 10). A surgeon was used in a combined function as triage responsible and medical leader. After the arrival of mobilised staff, the hospital had a maximum of 2 surgical consultants to advise the trauma teams and operate patients. Further, a maximum of 3 anaesthetic and 2 orthopaedic consultants were available during the test. Other professions assisted patient transport as there were a maximum of 3 porters available.

Voss municipality offered 50 nurses to support VH. Some of these nurses were put in duty for a 3-hour period. The HCG requested transfer of staff from HUH to support VH (5 vascular surgeons, 5 anaesthetic doctors, 3 anaesthetic nurses and 3 operating nurses arrived VH 9:51 pm).

#### Supplies

The HCG had regular meetings with HUH. Equipment was requested and transported to VH by ambulance (Table 6). Voss municipality donated 50 sets of patient clothing. VH had sufficient supplies of most equipment (Table 6). Before the first patient transfers to HUH, ICU had only one ventilator available. With a lower prehospital mortality rate or lack of patient transfers, the number of ventilators needed would have exceeded SC. The ED lacked a system for refilling the tray for chest drain insertion and the number of water locks was insufficient (Table 6). Additionally, there were limited quantities of pre-made major dressing kits, pelvic binders and tourniquets, but tourniquets and pelvic binders were also available prehospitally.

**Table 6:** Supplies

	Equipment	Number at Voss Hospital	Number ordered from Haukeland University Hospital	Number used	Comment
A/B	Laryngoscope	6	Not ordered	9	9 intubations in-hospital
	CMAC	3	Not ordered		
	Intubation tubes	> 100	9	9	9 intubations in-hospital
	Ventilators	9	5	9	9 intubations in-hospital
	Chest tube	> 100	Unknown order	17	1 prehospital tube inserted
	Chest tube water lock	6	Not ordered	18	
	Needle decompression	> 100	Not ordered	0	1 conducted prehospital
C	Tourniquet	2	Not ordered	0	1 applied prehospital
	Pelvic binder	3 sheet + 2 SAM	Not ordered	5	1 applied prehospital
	Blood infuser/warmer	8	Not ordered	8	11 patients received blood transfusion in-hospital
	Scoop	16	Not ordered		
	Portable blood pressure measure	16	Not ordered		
	Iv cannula	> 500	Not ordered	47	11 inserted prehospital
	Io cannula	34	Not ordered	1	
	Central line	50	Not ordered	3	
	ABG	> 100	Not ordered	15	
	Urinary catheter	> 100	Not ordered	8	
	Laparotomy instrument set	9	Not ordered	6	
	Vascular instrument set	0	5	0	
	Thoracotomy instrument set	2	Not ordered	0	
	External fixation set (orthopaedic)	3	Not ordered	0	1 Antebrachi set 1 Cruz set 1 Femur set
D/E	Neck collar	3 adult + 2 pediatric	Not ordered	1	1 applied prehospital
	Splint	6	Not ordered	3	3 applied prehospital
	Bed	103	Not ordered	Not exceeded	
	Transport scoop/stretcher	4	Not ordered	4	
	Major wound dressing kit (surgical)	8	Not ordered	9	Plenty of refill

#### Blood supply

The consultant on call at the blood bank (HUH) was informed early about a potential disaster (5:59 pm). The internal blood bank procedure for emergency collection of whole blood (“walking blood bank procedure”) was started 6:16 pm and 50 whole blood donations planned. The blood supply was discussed within the HCG 6:17 pm. The Blood Bank at HUH sent two loads of blood products with search and rescue helicopter (SAR) and HEMS.

In total, 13 patients received blood transfusion, 11 in-hospital. Four patients received blood prehospitally (4 units of whole blood). The blood usage was lower than expected based on the type and severity of injuries. The prehospital mortality (7/60, 11,7%) contributed to a lower-than-expected usage of blood transfusion in-hospital. Two patients needing a massive transfusion (MTP) to survive never arrived at VH. Of the patients transferred to HUH, one patient demanded MTP until definitive vascular repair. Four patients died due to internal bleeding after arrival at VH. One of them received 1 unit of lyophilised plasma at VH and one had received 1 unit of whole blood prehospitally. The clinicians were initially reluctant to order blood transfusion as the supply was regarded as insufficient. A lack of an established dynamic communication line regarding blood supply was detected. The availability of transfusion with platelet-containing blood products was limited.

#### Mortality

Delayed access of health services to the victims inside the tunnel resulted in 4 prehospital deaths. The test had a mortality rate of 14/60 (23,3%) and 9/14 (64,3%) of the deceased had ISS > 25 (Table 7). With the right optimal treatment, 10/14 (71,4%) of the patients could have survived and 5/14 (35,7%) with normal level of function. Two of the deceased had moderate injuries with ISS < 15 (Table 7). Bleeding was the main cause of death (Table 8). The triaged blue patient died in the ward due to flail chest/pulmonary contusion and lack of ventilator treatment.

**Table 7:** Mortality, injury severity score (ISS) and place of death

Location	Number of deaths	ISS < 15	ISS 15–25	ISS > 25
Incident site	7	1	2	4
Emergency department	4	1	0	3
Operating room	1	0	0	1
Ward	1	0	1	0
Haukeland University Hospital	1	0	0	1
Total	14	2	3	9

**Table 8:** Cause of death

	Bleeding	Lack of respiratory support	Tension pneumothorax	Head injury
Prehospital	2	3	2	0
In-hospital	4	1	1	1
Total	6	4	3	1

#### Evaluation of the test

A total of 91/98 (92,9%) of the participants responded to the survey. The hospital`s respondents found the MACSIM^®^ system useful to estimate SC (Table 9). Of the medical participants, 27/52 (52,9%) requested more time pre-test to learn the MACSIM^®^ system. Most medical participants ranked the “Vossa-test” high regarding improvement of the participants own role in an MCI/MI (Table 9).

**Table 9:** Evaluation of MACSIM (medical participants) (1 = not useful, 10 = very useful)

	Median	Min	Max	IQR
How would you rate the MACSIM as method to estimate the hospitals capacity in mass casualty incidents?	8 (*N* = 39)	3	10	1
How would rank Vossa-test as a tool to detect deficiencies in the hospital’s disaster preparedness?	8 (*N* = 40	3	10	1.75
How would you rank Vossa-test as a tool to improve your role in a mass casualty incident?	8 (*N* = 52)	2	10	3

*Note*: Different N due to questions regarding in−hospital conditions provided to hospital staff only and some questions provided to all medical staff (prehospital and in−hospital)

Participants regarded the wide range of participators as beneficial, and the scenario was ranked high (Table 10). Improved knowledge about the participants own role and tasks during an MCI/MI was reported by 76/91 (83,5%) and the participants evaluated the “Vossa-test” to be a valuable MCI/MI simulation exercise (Table 10).

**Table 10:** Evaluation of the “Vossa-test” (all participants) (1 = not useful, 10 = very useful)

	Median *N* = 91	Min	Max	IQR
How would you value the effect of including several organisations in an MCI/MI exercise as in the Vossa-test?	9	3	10	2
How would you rate the scenario in the Vossa-test?	8	2	10	3
How would you evaluate the Vossa-test as a simulation training	8	3	10	2.75

## Discussion

The methodology used was evaluated as a suitable tool to estimate SC and capacity-limiting factors at a small rural hospital. Inclusion of the most relevant civilian organisations for an MCI/MI response in the chosen scenario was valuable.

WHO recommends that hospitals “*calculate maximal capacity required for patient admission and care based not only on total number of beds required but also on availability of human and essential resources and the adaptability of facility space*” [24]. Studies indicate that there is a lack of knowledge regarding SC [25–27]. There is no consensus which method to apply for SC estimation [9, 17, 28–31]. Focusing on the numbers of beds and surgical teams alone is insufficient as organisation, communication and staff competency/skills are important factors [3–5, 32–35]. A full chain test method with figurants is resource demanding [36]. The MACSIM^®^ system can be used simultaneously with normal hospital activities.

MCI/MI bring inherent uncertainty, posing significant challenges to the organisations involved in the crisis management. Previous MCI/MI events have proven that cooperation and communication is of major importance [4, 33, 37–39]. The SC test was designed to address the importance of external cooperation and communication, factors shown to be important in disaster management at small hospitals [39, 40]. The broad inclusion of participating organisations was valued by the participants. A traffic scenario in MACSIM^®^ simulations had previously never been applied. Based on the feedback from the participants, the scenario was realistic. Rural hospitals face other challenges than larger hospitals when the disaster strikes [2, 32]. Limitations in terms of physical space, specialists, number of staff, equipment and blood supply are likely. Access to emergency medical transport resources is scarcer in rural areas [39]. Sarzynski et al. revealed concerns regarding disadvantages in transport availability at small rural hospitals during the Covid pandemic surge [41]. Hence, knowledge about the hospital`s SC is potentially more crucial at a rural hospital. The MACSIM^®^ method has previously been applied to assess SC at larger hospitals [16, 17]. Our study indicates that the method is valuable also for SC estimation at a small rural hospital. Cooperation with the TC was essential to enhance SC at VH. Based on the “Vossa-test”, TC participation in the SC test of a rural hospital could be recommended. In accordance with the Swedish study, requirements for increased pre-test time for the participants to learn the simulation system was detected [16]. A mandatory on-line course for participants introducing the MACSIM^®^ methodology could possibly enhance the participants pre-test skills.

Experiences from the “Twin attack” in Oslo 2011 showed that experienced surgeons in key functions were an important part of successfully managing the MCI [2, 32]. Correspondingly, VH placed a surgeon as triage responsible. In accordance with the computer models of Hirshberg et al., the lack of surgeons became the most obvious bottleneck in terms of SC at VH [42]. The number of major incident trauma teams was also a limiting factor in the Swedish SC tests [17]. In contrast to Ullevål Hospital (TC), Ringerike Hospital (NTC) experienced limitations in the number of available surgeons during the Utøya shooting in 2011 [2, 32]. Adaptions to the contingency plan regarding triage, trauma team composition and access to general surgeons had to be applied [2]. VH is considerably smaller than Ringerike Hospital. As a comparison, VH had 52 trauma alarms in 2022, whereas Ringerike Hospital had 254 [23]. These findings indicate that rural hospitals with limitations in crucial members of the trauma team must take this deficiency into consideration in the contingency planning. Small rural hospitals face the dilemma of placing surgeons in important roles as triage and medical leader role, which might critically reduce the number of available surgeons in the trauma teams and OR. The use of other trained staff in these functions should be considered.

Regarding physical space and patient flow, the pattern with ED receiving the peak first, followed by OR and ICU, correlated with the findings from the Swedish SC tests [17]. In contrast to the Swedish SC tests, the OR SC was reached at VH [17]. The time delay until the ICU peaks, needs to be acknowledged early to avoid exceeding capacity. Coordination and communication regarding patient referrals are crucial for hospitals with limited ICU capacity. Airborne traffic can be limited due to weather conditions, which must be taken into consideration in the contingency planning. Ward bed-capacity was sufficient and resembled previous MACSIM^®^ SC test results and experiences from MCI/MIs in Europe [5, 17, 43]. Access to CT scan limited the use of supplementary radiology in the test. Previous MACSIM^®^ SC tests have indicated that transfer of patients to CT locks up trauma team resources [17]. The use of CT scans in relation to the trauma reception was limited at both NTC and TC during the Utøya shooting in 2011, as opposed to the use of CT scanning for 83,8% of the trauma patients in Norway in 2022 [2, 23, 32].

Extra ventilators were requested early from HUH and the transfer of patients to HUH enhanced SC. The strategy of referral of ICU patients from NTC to TC was applied after the shooting at Utøya [2]. In the Las Vegas shooting, an improvised system with Y tubing was used to ventilate two persons with an estimated same size to compensate for a critical deficiency [44]. Despite larger bed-capacity in the Swedish tests, deficiency in supply of disposable surgical material and sets for external fixation were identified [17]. Sets for external fixation were limited at VH, but a plan to use one set on two patients was implemented. Only one orthopaedic surgery took place as laparotomies were prioritised. VH had insufficient quantities of water-lock systems for chest tubes, indicating the importance of running dynamic SC tests with inventory. Implementing a disaster storage with the necessary medical equipment to manage predefined critical events, based on risk analysis, could be an additional solution to enhance SC for supplies.

Early and balanced blood transfusions for patients with critical bleeding is crucial [45, 46]. Blood supply at VH is limited, and the simulation method gave detailed dynamic information about supplies and use of blood products. Many of the NTCs in Norway lack thrombocytes to provide an early and balanced blood transfusion, as recommended in the national trauma plan [22]. VH has since 2019 had 2 whole bloods in the stock [47]. Blood supply was early on the agenda during the test, and two loads of blood supply was transferred from HUH. The blood bank at HUH has been an active part of whole blood implementation in Norway [47–49]. The test detected that there was a discrepancy between the actual blood supply and the clinician’s assumption. Fear of running out of blood prevented some of the first arriving patients to be transfused. Implementing good routines regarding dynamic overview of the blood supply at the hospital is an important area of improvement. Establishing whole blood preparedness through walking blood bank is feasible and should be investigated as a strategy for VH, both for a single patient requiring massive transfusion protocol or in an MCI/MI situation [49–52].

Victim identification was detected as an area of improvement. The list of fatalities after the terrorist attack in Norway in 2011 was released to the media 4 days after the attack, although the forensic identification was not completed until 5 days after that [53]. The 22nd of July commission stated that the collaboration between health and police regarding information and following-up routines of relatives should be considered [53]. The identification process is composed of a forensic part involving the deceased, but also include patients that are unable to identify themselves [54]. Lessons learned from international events show that delays in the identification process impose concerns among relatives [33, 55].

### Limitations

As all simulations, the “Vossa-test” carries the limitation of not representing reality and a real event. Although, a notable number of details were provided, corresponding to a large degree with results from similar MACSIM^®^ SC tests and experiences from real events. The MACSIM^®^ simulation has limitations in assessing the actual available physical spaces. The use of complementary simulation methods such as in situ figurant exercises, where stretches, beds and people are moved, is important. In future MACSIM^®^ SC tests involving a rural hospital, it would be of interest to include the TC with all relevant wards as participants. A different scenario with Hazmat and burns would most likely result in a different patient distribution from the scene. Developing a digital solution replacing the protocol writers, scanning the treatment tags when leaving each test station and providing immediate test data for analysis would be an area of improvement. This would probably make the SC test easier to plan, conduct and evaluate.

## Conclusions

The simulation system was found useful as an SC test tool at a small rural hospital, providing detailed data in terms of system, space, staff and supplies. The methodology could also be applied as a tool to enhance staff competency in MCI/MIs. Developing methods to promote the participants pre-test knowledge regarding the simulation system would be beneficial.

## Electronic supplementary material

Below is the link to the electronic supplementary material.

Supplementary Material 1

Supplementary Material 2

## Data Availability

No datasets were generated or analysed during the current study.

## Abbreviations

CBRNE: Chemical, Biological, Radiological, Nuclear, Explosive
ED: Emergency department
HCG: Hospital command group
HEMS: Helicopter emergency medical service
HGV: Heavy goods vehicle
HUH: Haukeland University Hospital
ICU: Intensive care unit
ISS: Injury severity score
MACSIM: Mass Casualty Simulation System
MCI: Mass casualty incident
MI: Major incident
MRMI: Medical Response to Major Incident
MTP: Massive transfusion protocol
NTC: Non-trauma centre/ acute hospital with trauma function
OOH: Out-of-hours emergency primary health care
OR: Operating room
SAR: Search and rescue helicopter
SC: Surge capacity
TC: Trauma centre
VH: Voss Hospital
