# Supplementary material for: A full response chain surge capacity test of a small rural hospital, prehospital resources and collaborating organisations
Source: Scand J Trauma Resusc Emerg Med. 2025 Mar 28;33:55. doi: 10.1186/s13049-025-01372-9 (PMC11954251; doi:10.1186/s13049-025-01372-9)
Supplement: Supplementary file 1 — Supplementary Material 1 [file 13049_2025_1372_MOESM1_ESM.docx]

**Appendix**  Survey «Vossa-test»

1. Which organisation do you work for?

○ Do not want to answer

○ Voss Hospital-Not Hospital Command Group

○ Voss Hospital- Hospital Command Group

○ Police

○ Fire and Rescue

○ Civil Protection

○ Out-of hours Primary Health Care

○ Ambulance

○ Helicopter Emergency Medical Service

○ Emergency Medical Communication Centre (113)

○ Road Authority

○ Road Information Central

○ Other: (Specify)

○ Red Cross

○ Police Operation Central (110)

○ Fire Operational Central (112)

○ Voss Municipality-disaster Comittee

1. How would you evaluate the MACSIM methods relevancy in in-hospital Surge Capacity estimation in a Mass Casualty Incident? (1=not suitable, 10=very suitable)

○ 1

○ 2

○ 3

○ 4

○ 5

○ 6

○ 7

○ 8

○ 9

○ 10

1. How useful do you considered the Vossa-test is to detect potential deficiencies or areas of improvement in the hospital disaster preparedness? (1=not suitable, 10=very suitable)

○ 1

○ 2

○ 3

○ 4

○ 5

○ 6

○ 7

○ 8

○ 9

○ 10

1. To what degree did the Vossa-test improve your knowledge regarding your role in a Mass Casualty Incident? (1=not suitable, 10=very suitable)

○ 1

○ 2

○ 3

○ 4

○ 5

○ 6

○ 7

○ 8

○ 9

○ 10

1. Would you want more time to learn the MACSIM method before the Vossa-test?

○ Yes

○ No

○ Did not know

1. If yes on question 5. How much time do you consider reasonable?
2. How well were you aware of the hospital’s alertness level for disaster BEFORE the Vossa-test?

○ Unknown

○ Heard of it, but uncertain about what the different levels meant

○ Good knowledge regarding different alertness levels (green, yellow, red)

1. How well did you know the hospital’s contingency plan for Mass Casualty Incident BEFORE the Vossa-test?

○ Never seen the contingency plan

○ Had seen the plan and knew where to find it, but have not read it

○ Read it long time ago

○ Recently read the contingency plan and had good overview over the parts relevant for my own function/role

1. How often is there a joint review of the contingency plan for Mass Casualty Incident at your workplace?

○ Never

○ Sometimes, not regularly

○ Yearly review of up-dates/changes

1. Did your organisation apply the contingency plan for Mass Casualty Incidents during the Vossa-test?

○ Yes

○ No

○ Did not know

1. How do you consider your organisations contingency plan for Mass Casualty Incidents performed during the Vossa-test?

○ Performed well

○ Performed, but need of revision

○ Did not perform well, need of larger revision

1. Is there an action card for the role/function you had during the Vossa-test?

○ Yes

○ No

○ Did not know

1. If yes on question 12, how did your action card work?

○ Never followed the action card

○ Partially followed the action card, but the action card needs revision

○ Followed the action card, the action card worked well

1. Which education/courses/training have you received to perform your tasks in a Mass Casualty Incident like the scenario at the Vossa-test?

○ None

○ Cours in the basic education

○ Post graduate course

○ Voluntary post graduate course

○ Education/training provided by the employer

○ Exercise provided by the employer

1. Do you consider the education/training you have received sufficient to perform well in an Incident of this severity (Vossa-test)?

○ Insufficient

○ Useful, but would have needed more

○ Sufficient

1. How well were you prepared to work with a Mass Casualty like Vossa-testen? (1=not prepared, 10=very prepared)

○ 1

○ 2

○ 3

○ 4

○ 5

○ 6

○ 7

○ 8

○ 9

○ 10

1. Give your impression of how your organisation as a whole performed during Vossa-testen (1=insufficient, 10=very well)

○ 1

○ 2

○ 3

○ 4

○ 5

○ 6

○ 7

○ 8

○ 9

○ 10

1. How do you evaluate the internal communication within you organisation during the Vossa-test? (1=insufficient, 10=very well)

○ 1

○ 2

○ 3

○ 4

○ 5

○ 6

○ 7

○ 8

○ 9

○ 10

1. How do you evaluate the communication between organisation during the Vossa-test? (1=insufficient, 10=very well)

○ 1

○ 2

○ 3

○ 4

○ 5

○ 6

○ 7

○ 8

○ 9

○ 10

1. How do you evaluate the collaboration between the different participating organisation during the Vossa-test? (1=insufficient, 10=very well)

○ 1

○ 2

○ 3

○ 4

○ 5

○ 6

○ 7

○ 8

○ 9

○ 10

1. How do you evaluate the benefit of exercising several prehospital and inhsopital actors as in the Vossa-test? (1=not useful, 10=very useful)

○ 1

○ 2

○ 3

○ 4

○ 5

○ 6

○ 7

○ 8

○ 9

○ 10

1. Have you previously participated in a multidisciplinary table-top with more than 3 participating organisations/actors?

○ Yes

○ No

○ Did not know

1. Did the participation in Vossa-test improve your knowledge about your own tasks and role/function in an disaster event?

○ Yes

○ No

○ Did not know

1. How would you evaluate the scenario in the Vossa-test? (1=unrealistic, 10=very realistic)

○ 1

○ 2

○ 3

○ 4

○ 5

○ 6

○ 7

○ 8

○ 9

○ 10

1. How would you evaluate the Vossa-test as simulation training for Mass Causualty Incidents? (1=not suitable, 10=very suitable)

○ 1

○ 2

○ 3

○ 4

○ 5

○ 6

○ 7

○ 8

○ 9

○ 10

1. Alarming: Learning points/your comments from the Vossa-test
2. Leadership: Learning points/your comments from the Vossa-test
3. Organisation: Learning points/your comments from the Vossa-test
4. How would you evaluate the joint evaluation after the Vossa-test at Voss Cinema?

○ Did not participate

○ 1

○ 2

○ 3

○ 4

○ 5

○ 6

○ 7

○ 8

○ 9

○ 10

1. Comments/Suggestions for improvement
