# Supplementary material for: A full response chain surge capacity test of a small rural hospital, prehospital resources and collaborating organisations
Source: Scand J Trauma Resusc Emerg Med. 2025 Mar 28;33:55. doi: 10.1186/s13049-025-01372-9 (PMC11954251; doi:10.1186/s13049-025-01372-9)
Supplement: Supplementary file 2 — Supplementary Material 2 [file 13049_2025_1372_MOESM2_ESM.pptx]

## Slide 1
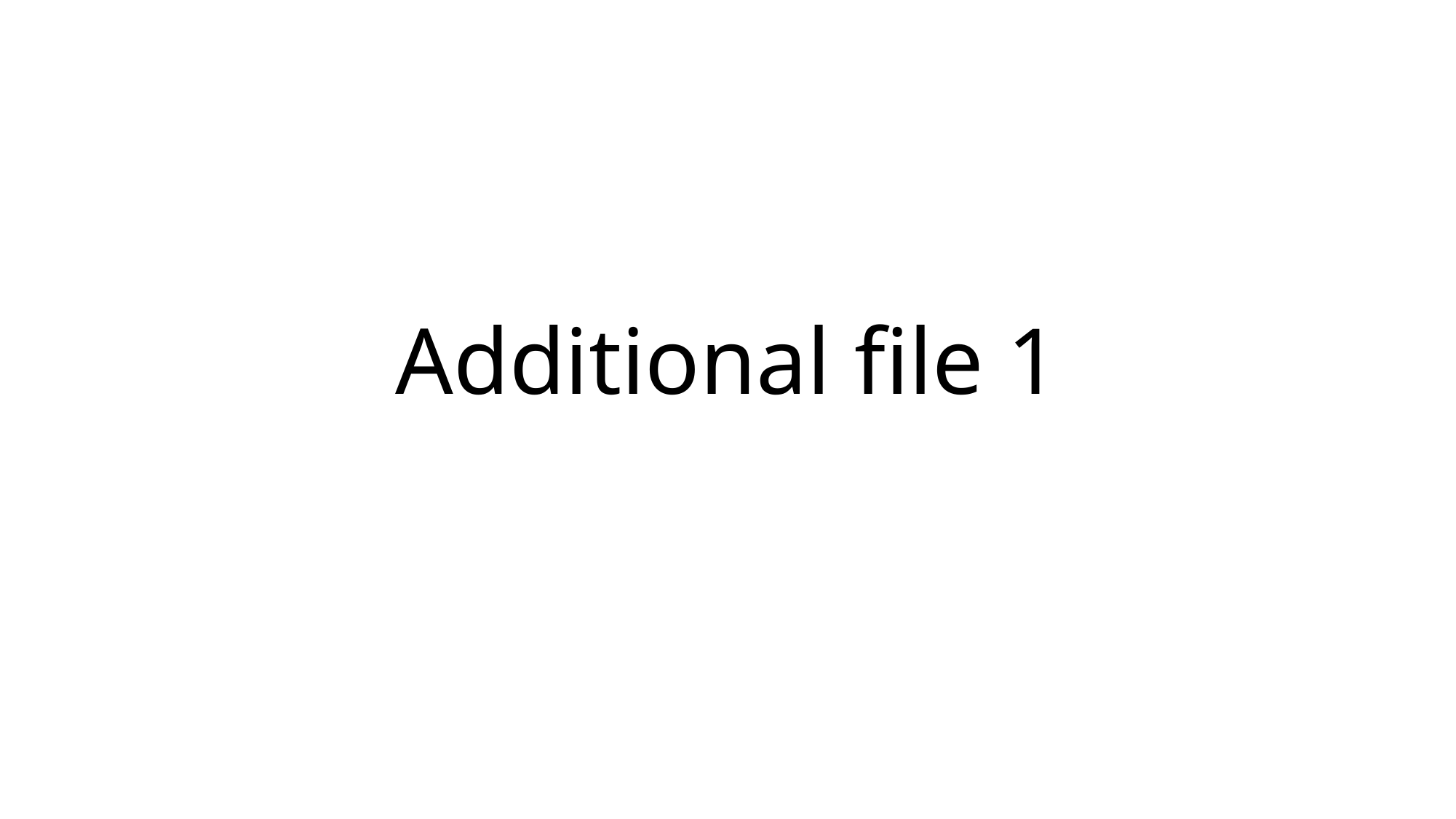

# Additional file 1

## Slide 2
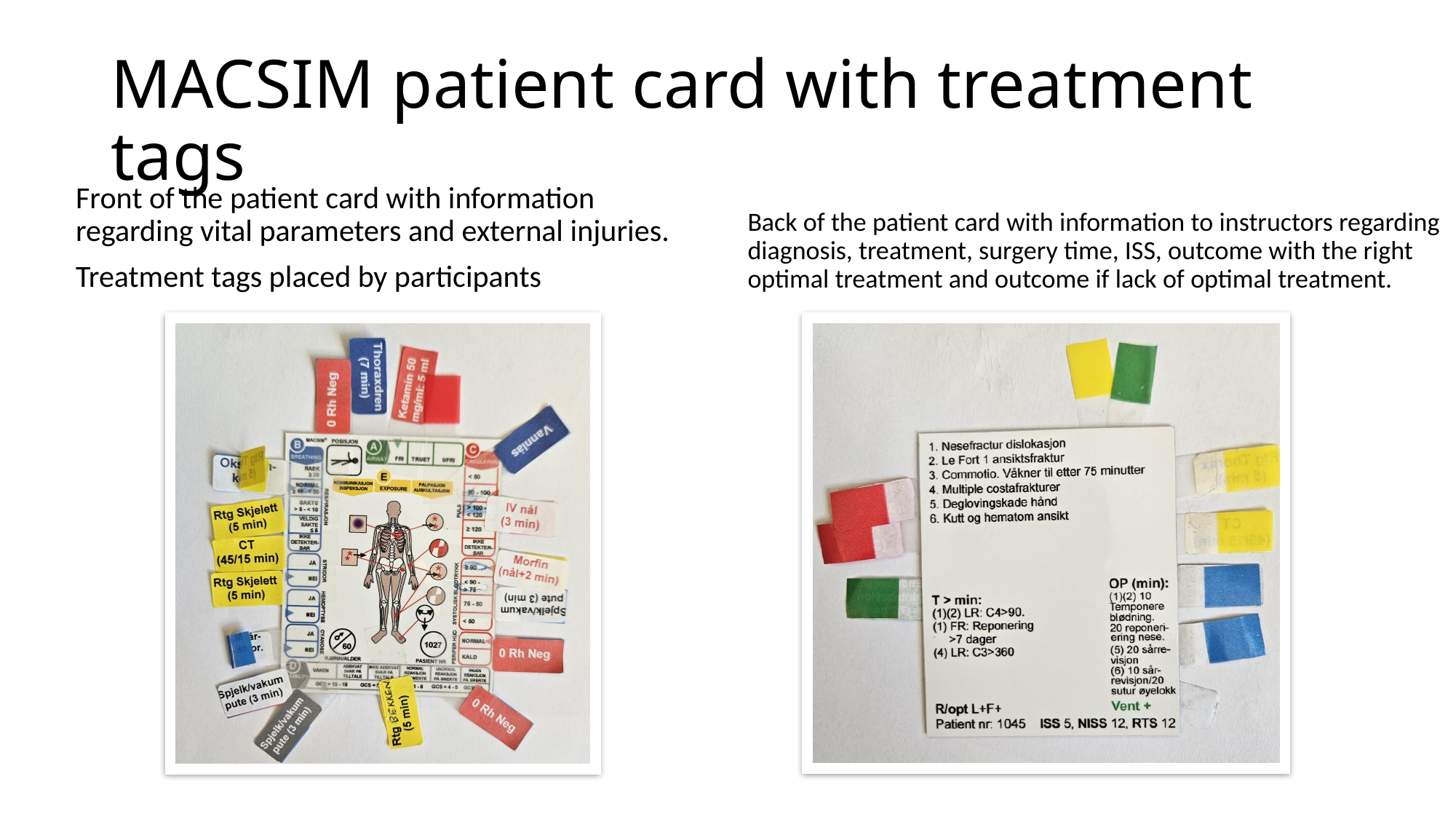

# MACSIM patient card with treatment tags
Front of the patient card with information regarding vital parameters and external injuries.
Treatment tags placed by participants
Back of the patient card with information to instructors regarding diagnosis, treatment, surgery time, ISS, outcome with the right optimal treatment and outcome if lack of optimal treatment.

## Slide 3
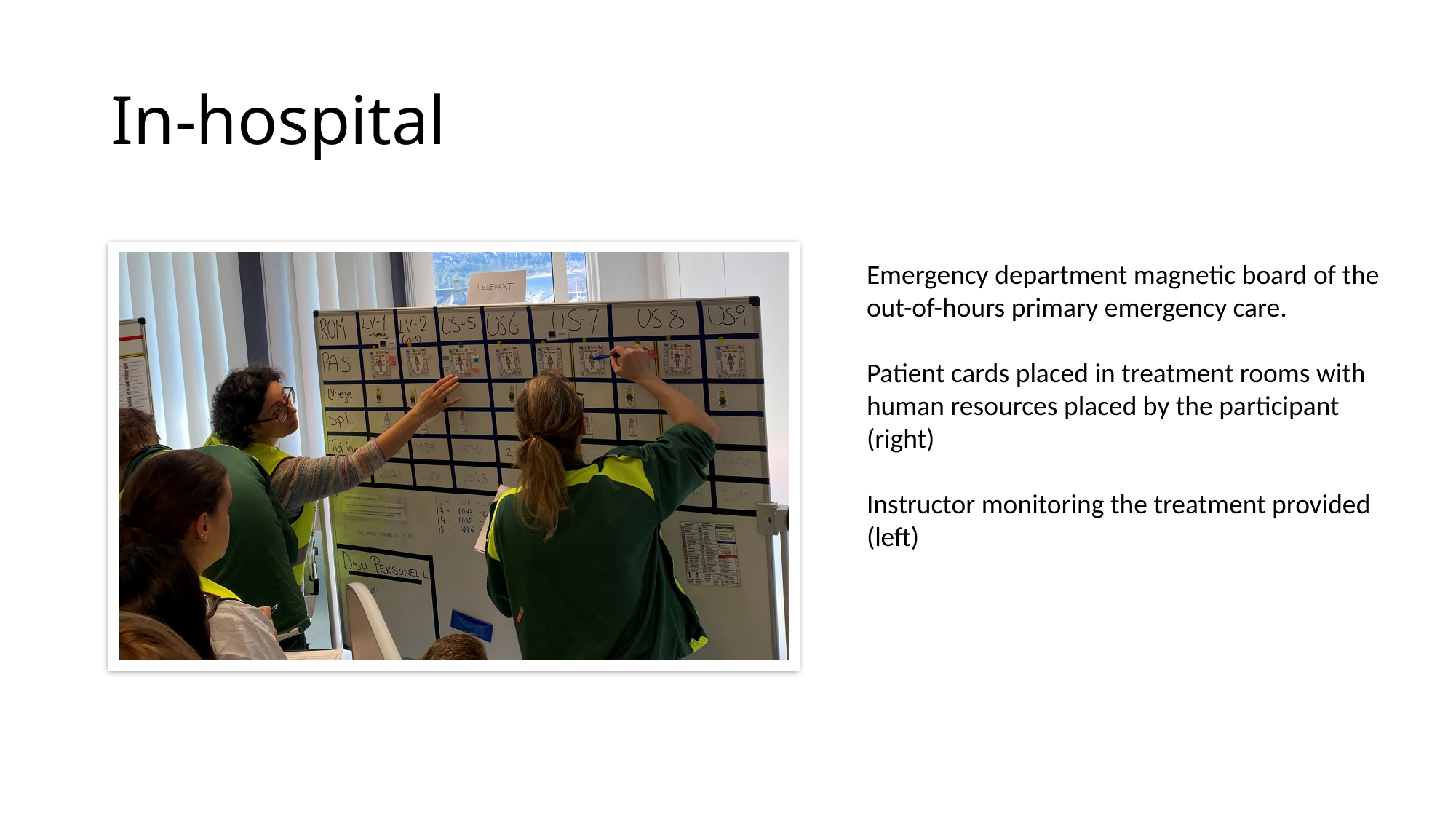

# In-hospital
Emergency department magnetic board of the out-of-hours primary emergency care.
Patient cards placed in treatment rooms with human resources placed by the participant (right)
Instructor monitoring the treatment provided (left)

## Slide 4
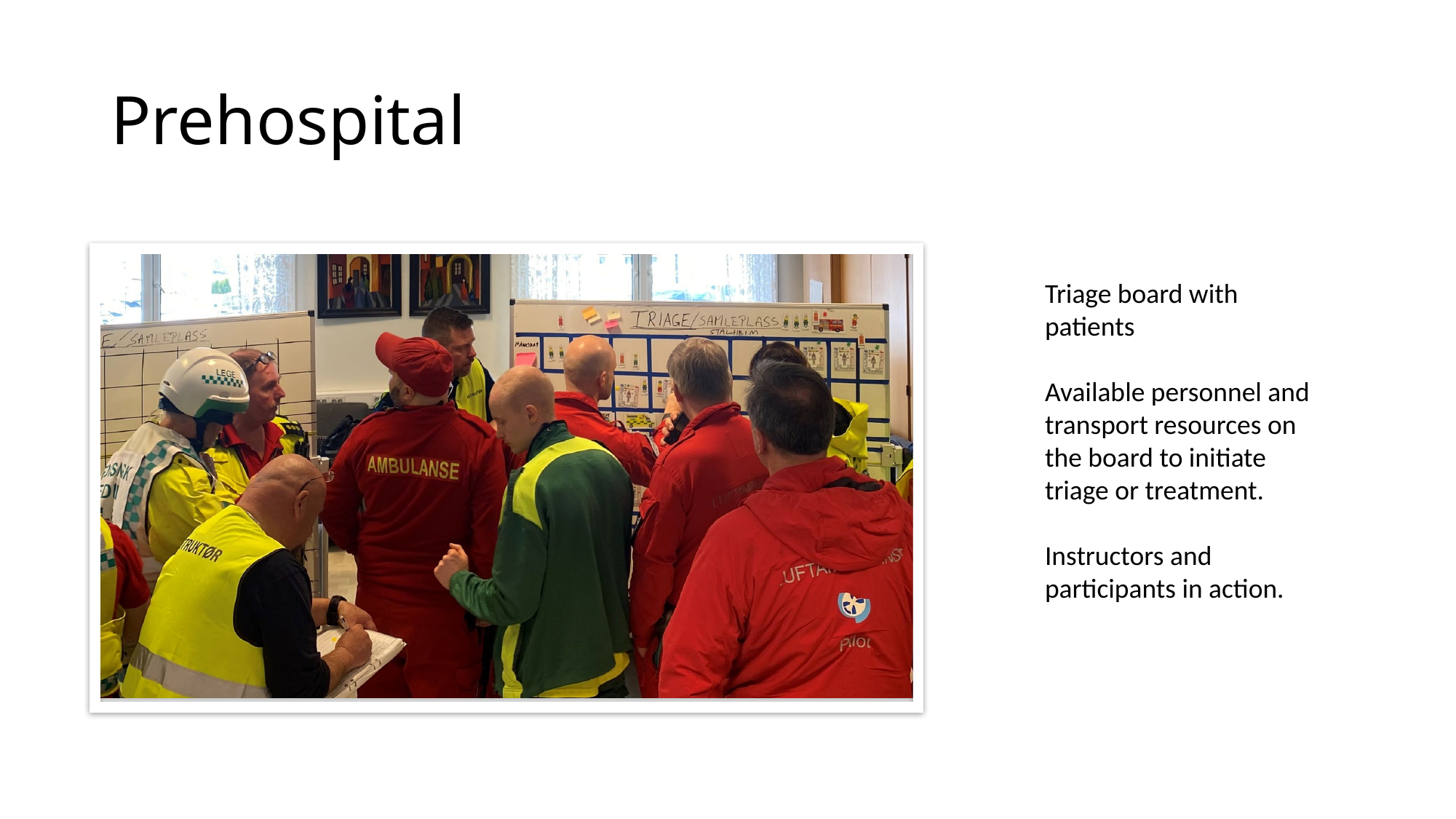

# Prehospital
Triage board with patients
Available personnel and transport resources on the board to initiate triage or treatment.
Instructors and participants in action.
